# The MET Oncogene Network of Interacting Cell Surface Proteins

**DOI:** 10.3390/ijms252413692

**Published:** 2024-12-21

**Authors:** Simona Gallo, Consolata Beatrice Folco, Tiziana Crepaldi

**Affiliations:** 1 Department of Oncology, University of Turin, Regione Gonzole 10, 10143 Orbassano, Italy; simona.gallo@unito.it (S.G.); consolatabeatrice.folco@unito.it (C.B.F.); 2 Candiolo Cancer Institute, FPO-IRCCS, SP142, Km 3.95, 10060 Candiolo, Italy

**Keywords:** HGF/MET receptor axis, receptor tyrosine kinases, co-receptors, adhesion molecules, proteases, combination therapies

## Abstract

The MET oncogene, encoding the hepatocyte growth factor (HGF) receptor, plays a key role in tumorigenesis, invasion, and resistance to therapy, yet its full biological functions and activation mechanisms remain incompletely understood. A feature of MET is its extensive interaction network, encompassing the following: (i) receptor tyrosine kinases (RTKs); (ii) co-receptors (e.g., CDCP1, Neuropilin1); (iii) adhesion molecules (e.g., integrins, tetraspanins); (iv) proteases (e.g., ADAM10); and (v) other receptors (e.g., CD44, plexins, GPCRs, and NMDAR). These interactions dynamically modulate MET’s activation, signaling, intracellular trafficking, and degradation, enhancing its functional versatility and oncogenic potential. This review offers current knowledge on MET’s partnerships, focusing on their functional impact on signaling output, therapeutic resistance, and cellular behavior. Finally, we evaluate emerging combination therapies targeting MET and its interactors, highlighting their potential to overcome resistance and improve clinical outcomes. By exploring the complex interplay within the MET network of interacting cell surface proteins, this review provides insights into advancing anti-cancer strategies and understanding the broader implications of RTK crosstalk in oncology.

## 1. Introduction

The MET receptor, a receptor tyrosine kinase (RTK), plays a central role in cellular processes such as proliferation, migration, survival, and angiogenesis. Its dysregulation is a hallmark of numerous cancers, contributing to tumor progression, metastasis, and resistance to therapies. Despite extensive studies on MET and its primary ligand, hepatocyte growth factor (HGF), several critical gaps in our understanding remain. This review seeks to address these gaps by focusing on (i) the molecular mechanisms underpinning MET signaling activation. While the structural dynamics of MET activation by HGF are well characterized, the nuances of its dimerization, the influence of cofactors such as heparin, and the stabilization of receptor–ligand complexes have been recently elucidated by cryo-microscopy studies. Understanding these processes at a molecular level is essential to refine therapeutic strategies that target MET signaling; (ii) the interactions of MET with other membrane proteins and their implications in cancer modulate MET signaling pathways, often amplifying its oncogenic potential. The mechanisms by which these protein complexes influence MET function, particularly in resistance to targeted therapies, is still underexplored. This review provides a comprehensive analysis of MET’s interactome, highlighting its role in cancer progression and therapy resistance; (iii) current MET-targeted therapies face challenges due to the receptor’s ability to form compensatory signaling networks. This review explores emerging combination therapies that simultaneously target MET and its interactors, aiming to overcome resistance mechanisms and improve clinical outcomes. Additionally, novel therapeutic strategies targeting MET-driven metastasis and cancer stem cells are discussed, offering insights into innovative approaches to combat MET-related oncogenesis.

## 2. Structure of MET

**Figure 1:**
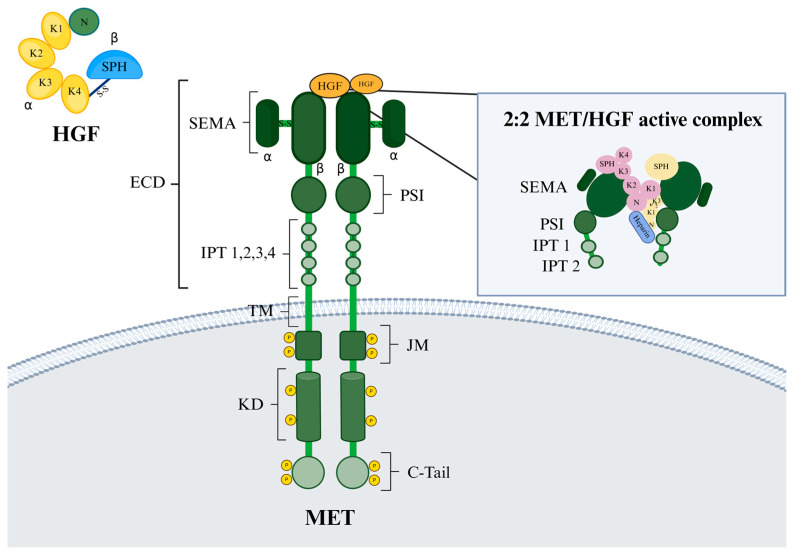
Molecular structure of MET receptor and its natural ligand HGF. C-tail: C-terminal tail; ECD: extracellular domain; IPT: immunoglobulin–plexin–transcription factor domain; JM: juxta membrane domain; K1–K4: kringle domains; KD: kinase domain; N: N-terminal domain; PSI: plexin–semaphorin–integrin domain; SEMA: semaphorin-like domain; SPH: serine protease homology domain; TM: transmembrane domain. Inset: 2:2 MET-to-HGF active complex. Created in BioRender (accessed on 18 December 2024).

The human MET is encoded by the *MET* gene, located on chromosome 7, band 7q21-q31, spanning over 120 kb [1]. In wild-type cells, MET transcription produces a 150 kDa polypeptide that undergoes partial glycosylation to form a 170 kDa-precursor protein [2]. This precursor is then further glycosylated and, upon cleavage by the furin protease between Arg307 and Ser308, the mature MET receptor is generated. The mature form consists of two subunits—the 50 kDa α subunit and the 145 kDa β subunit—linked by at least three disulfide bonds to form the cell surface receptor. Similar to other receptor tyrosine kinases (RTKs), the final structure of MET comprises different regions (Figure 1). 

The extracellular domain (ECD) is exposed on the cell surface and is responsible for ligand binding. The transmembrane domain (TM) is a single membrane-spanning segment anchoring the receptor to the cell membrane. The juxtamembrane domain (JM), located adjacent to the transmembrane region, plays a role in negatively regulating receptor activity. The kinase domain (KD), upon dimerization, catalyzes the trans-autophosphorylation of tyrosine residues. Lastly, the C-terminal tail (C-tail) serves as a docking site for adaptor proteins, enhancing downstream signaling.

The ECD of MET contains several distinct domains: a SEMA domain (semaphorin-like domain), a PSI domain (plexin–semaphorin–integrin domain), and four IPT domains (immunoglobulin–plexin–transcription factor domain) (Figure 1) [3]. The SEMA domain is structurally similar to semaphorins and critical for ligand binding. The PSI domain (plexin–semaphorin–integrin) aids in receptor stability. IPT domains (immunoglobulin–plexin–transcription factor) contribute to ligand interaction and receptor dimerization. The ligand of MET, the hepatocyte growth factor (HGF), is encoded by its homonymous gene located on chromosome 7q21.1, and is initially transcribed as an inactive single-chain precursor, pro-HGF, with a molecular weight of 90 kDa. This precursor contains an N-terminal (N) domain, four consecutive kringle (K1–K4) domains, and a serine protease homology (SPH) domain. The SPH domain resembles serine proteases but lacks enzymatic activity. To produce the active form of HGF, pro-HGF undergoes proteolytic cleavage between residues Arg494 and Val495, yielding two subunits, α and β, with molecular weights of 60 and 32 kDa, respectively (Figure 1). These subunits are then linked by a dilsufide bond between the Cys487 of the α-subunit and the Cys604 of the β subunit. This proteolytic cleavage is essential for MET activation: while both pro-HGF and cleaved HGF bind the receptor with high affinity, only the cleaved form of HGF can activate the downstream MET signaling pathway [4].

Cryo-electron microscopy (cryo-EM) has revealed the three-dimensional structure of active HGF bound to MET (SEMA-PSI-IPT1-IPT2) at near-atomic resolution [5,6]. This analysis showed that one ligand molecule (HGF) binds to and activates two MET receptor molecules, facilitating dimerization and signal transduction. The 2:1 receptor-to-ligand activation model, also known as the “single ligand-induced dimerization model”, has been observed in the activation of other receptors, such as the growth hormone receptor from the class I cytokine receptor family. This model represents a unique mechanism underlying activation within the RTK family [6]. Although one HGF molecule is sufficient for receptor activation, the MET-to-HGF complex structure reveals an asymmetric 2:2 MET-to-HGF assembly (Figure 1, inset). Specifically, the minimal 2:1 MET-to-HGF active complex is further stabilized by a second HGF molecule and heparin, resulting in a more stable 2:2 complex and enhanced MET activation. As previously mentioned, a single HGF molecule, when elongated, can bridge two MET receptors at opposite ends. In interacting with the first MET molecule, HGF engages the SEMA domain of MET at four distinct interfaces involving the N, K2, K3, and SPH domains. On the opposite side, HGF makes direct contact with the second MET molecule solely through its K1 domain, highlighting the essential role of this domain in MET activation. Heparin contributes to this interaction by binding the N domain of HGF and the IPT1 domain of one of the two METs, thus enhancing the high-affinity association between these molecules. Furthermore, a second HGF molecule stabilizes the overall complex by contacting the SEMA and PSI domains of the second MET molecule through its SPH and K1 domains, respectively. It also weakly contacts the N domain of the first HGF molecule via its K1 and K3 domains, resulting in a more stable 2:2 MET-to-HGF holo-complex [5].

## 3. Cell Surface Proteins Interacting with MET

MET’s function and regulation are significantly influenced by its interactions with other membrane-spanning molecules, which modulate its signaling pathways and impact cellular outcomes. Among the membrane-spanning partners of the MET receptor, we will focus on RTKs, co-receptors, like CDCP1 and Neuropilin1, adhesion molecules such as integrins and tetraspanins, proteases, like ADAM10, and other receptors, such as CD44, plexins, GPCRs, and NMDAR (Table 1).

### 3.1. Receptor Tyrosine Kinases

MET interacts with Recepteur d’Origine Nantais (RON), Receptor Tyrosine Kinase-Like Orphan Receptor 1 (ROR1), the human epidermal growth factor receptor (HER) family, which includes EGFR/HER1, HER2/ErbB2, and HER3/ErbB3, the vascular endothelial growth factor receptor (VEGFR), and several other RTKs, forming complex signaling networks that influence tumor biology and therapy resistance.

#### 3.1.1. MET-RON Interaction

**Figure 2:**
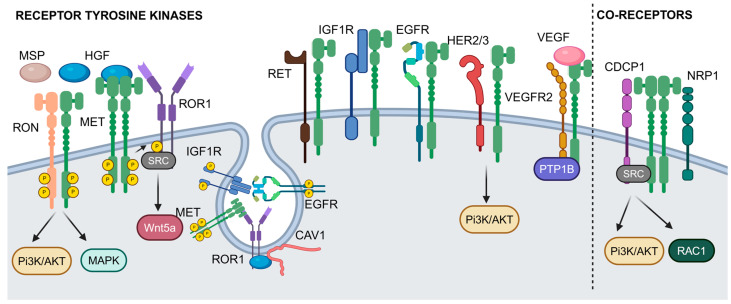
Cell surface proteins interacting with MET: receptor tyrosine kinases and co-receptors. Created in BioRender (accessed on 18 December 2024).

MET and RON share structural domains (SEMA, PSI, and IPT domains) but engage distinct ligands (HGF for MET, and MSP for RON) [95]. Their heterodimerization results in transphosphorylation, activating pathways like PI3K/AKT and MAPK [7,8], which enhance tumor growth, chemoresistance, and poor prognosis [9,10]. Co-targeting MET and RON offers a promising strategy for tumors co-expressing these RTKs (Figure 2).

#### 3.1.2. MET-ROR1 Crosstalk

MET phosphorylates ROR1 (Figure 2) [11,12], which is overexpressed in certain cancers, particularly B-cell malignancies [96,97,98]. This phosporylation in the proline-rich domain (PRD) of ROR1 recruits Src, a non-receptor tyrosine kinase, which leads to further phosphorylation within the ROR1 kinase domain and activates Wnt5a signaling [96]. Additionally, MET is known to be expressed in various B-cell malignancies [99], highlighting the potential for targeting MET and ROR1 in tandem. In lung adenocarcinoma, ROR1 acts as a scaffold for cavin-1 and caveolin-1 (CAV1), two proteins critical for caveolae formation and function. Caveolae are small invaginations in the cell membrane involved in signal transduction [100]. The interaction of cavin-1 and CAV1 at the plasma membrane protects CAV1 from lysosomal degradation, stabilizing AKT-mediated survival pathways stimulated by multiple RTKs, including MET. This mechanism explains how inhibiting ROR1 can help overcome the resistance to EGFR-tyrosine kinase inhibitors (TKIs) by blocking bypass signaling through alternative RTKs like MET and the insulin-like growth factor-I receptor (IGF1R). Given ROR1’s onco-embryonic expression, targeting its scaffold function presents a promising strategy for improving treatment outcomes in lung adenocarcinoma.

#### 3.1.3. MET-EGFR and HER Family Interactions

The MET-EGFR crosstalk is one of the most studied RTK interactions, particularly in cancer drug resistance. The EGFR inhibition by cetuximab can lead to a compensatory activation of MET, thereby driving resistance to targeted therapies (Figure 2) [13,14,15,16,17]. In head and neck cancer (HNC), HGF primarily functions as a paracrine factor, secreted by cancer-associated fibroblasts, making it prevalent in the tumor micro-environment. While this paracrine effect is the main mode of MET activation, MET can also be activated independently of its ligand through Src mediation, especially in tumors resistant to EGFR inhibitors (e.g., erlotinib or gefitinib) [101,102]. Additionally, MET activation can occur via heterodimerization with HER3 [13,26,27]. Interestingly, a MET-positive gastric carcinoma cell line became resistant to MET inhibitors through bypass activation of the EGFR pathway [103]. This reciprocal relationship between MET and EGFR highlights the complexity of RTK signaling networks and underscores the need for combination therapies to effectively overcome resistance mechanisms.

The interaction between MET and EGFR significantly contributes to the progression and drug resistance in non-small cell lung carcinoma (NSCLC). EGFR signaling promotes MET phosphorylation, which, along with the co-expression of HER3, enhances receptor activation. This crosstalk involves the regulation of MET levels and intermediate signaling through MAPK pathways. In NSCLC, with either wild-type or mutant EGFR, inhibiting EGFR or MAPK reduces MET activation and protein levels, highlighting the critical role of EGFR-MET signaling in the aggressive behavior of NSCLC and its potential as a therapeutic target [18].

The discovery that 22% of lung cancer specimens resistant to gefitinib or erlotinib show MET amplification points to a significant mechanism of drug resistance [19,20,21]. MET amplification leads to HER3-dependent PI3K activation, a pathway typically associated with the EGFR/ErbB/HER family. This suggests that MET’s role in resistance extends beyond individual receptors, potentially affecting a range of ErbB-driven cancers, and underscores the necessity of targeting this pathway in combination treatment strategies [19,22]. Among the EGFR family, HER3 shows a notable preference for MET-dependent trans-phosphorylation, underscoring the idea that the kinase activity of MET is driving the phosphorylation of kinase-dead ErbB3 receptors [28]. MET amplification frequently emerges as a mechanism of acquired resistance in various oncogene-driven molecular subsets of NSCLC following tyrosine kinase inhibitor treatment [23].

A recent study examined the effects of single MET inhibition in EGFR-mutant and MET-amplified NSCLC [24]. It found that single MET inhibition effectively suppresses EGFR downstream signaling and proliferation in specific lung cancer cells. However, the response was not durable, suggesting that further investigation into novel combination therapies is needed to achieve longer-lasting efficacy with minimal toxicity.

#### 3.1.4. VEGFR and MET Crosstalk

In glioblastoma multiforme (GBM), the vascular endothelial growth factor (VEGF) suppresses tumor cell invasion by promoting the recruitment of protein tyrosine phosphatase 1B (PTP1B) to a complex formed by MET and VEGF receptor 2 (VEGFR2, Figure 2) [30]. PTP1B is an enzyme that dephosphorylates tyrosine residues on receptors like MET, acting as a regulator to prevent the overactivation of signaling pathways. This interaction inhibits the phosphorylation of MET. Consequently, the inhibition of VEGF signaling, particularly using bevacizumab (a VEGF-blocking antibody), disrupts this suppressive effect. As a result, MET activity is restored and even enhanced in GBM cells, leading to increased invasion capabilities. Dual inhibition of VEGF and MET provides a significant survival benefit in GBM models, highlighting a potential therapeutic strategy for combating the invasive properties of GBM in patients treated with VEGF inhibitors like bevacizumab.

#### 3.1.5. Broader RTK Interactions

MET also interacts with the rearranged during transfection (RET) receptor, IGF1R, and other RTKs (Figure 2) [25,29,104], contributing to cell proliferation, migration, and survival. These partnerships result in distinct functional outcomes, such as the migration driven by RET or survival mediated by IGF1R [25]. Using affinity purification coupled to mass spectrometry, Salokas et al. [31] identified significant interactions also between MET and the neurotrophic receptor tyrosine kinase 3 (NTRK3), platelet-derived growth factor receptor β (PDGFRβ), insulin receptor (INSR), and tyrosine-protein kinase receptor (TYRO3). The co-option of multiple RTKs highlights the complexity of MET’s role in oncogenesis and resistance mechanisms, necessitating combination therapies.

### 3.2. Co-Receptors

#### 3.2.1. The Transmembrane Activator of Src CDCP1

Gusenbauer et al. (2013) [32] identified, via mass spectrometry, novel binding partners of TK-inactive EGFR, including AXL and EphA2 RTKs, and CDCP1 (CUB domain-containing protein), highlighting their roles in cancer progression. CDCP1 physically interacts with MET (Figure 2), particularly in contexts like squamous cell carcinoma, where this interaction contributes to resistance to EGFR inhibitors and promotes tumor growth, metastasis, and therapy resistance. Osimertinib, a third-generation TKI, irreversibly targets specific EGFR mutations [105]. In osimertinib-resistant lung cancer with T790M mutations, the most common mechanism of acquired resistance developing after treatment with first-generation EGFR TKIs, a significant reduction in the expression of multiple EGFR family proteins and MET, was observed [33]. Conversely, there was an increased expression of AXL, CDCP1, and SRC, along with enhanced AKT activation. Silencing both CDCP1 and AXL increased the sensitivity of cells to osimertinib. Additionally, inhibiting SRC, either through silencing or with the SRC family kinase (SFK) inhibitor dasatinib, reduced AKT phosphorylation and inhibited cell growth [33]. The CDCP1-SRC axis is also crucial for HGF and ARHGEF7-RAC1 signaling in MDA-MB-231 cells [34], contributing to epithelial-mesenchymal transition (EMT), and bypass resistance mechanisms.

#### 3.2.2. Neuropilin-1

Neuropilin-1 (NRP1) is a transmembrane glycoprotein that contributes to the growth and metastasis of cancer cells by acting as a multifunctional co-receptor and engaging with various signaling pathways (Figure 2) [106]. NRP1 was initially described as a co-receptor for secreted semaphorins and for VEGFs. Additionally, the extracellular domain of NRP1 interacts with EGFR, enhancing its signaling cascade triggered by EGF or TGF-α stimulation [107]. When NRP1 is silenced, the ability of ligand-bound EGFR to cluster on the cell surface, undergo internalization, and activate the downstream AKT pathway, is significantly compromised [107]. In pancreatic cancer cells, which express relatively high levels of NRP1, the suppression of endogenous NRP1 completely abolished HGF-mediated cell invasion [35]. The combination of NRP1-interfering molecules with MET-targeted drugs enhanced their effectiveness and prevented, or even reversed, the development of resistance in cancer cells and tumor models [36]. Consistent with other findings linking MET and EGFR oncogenic signaling, the resistance to MET inhibitors in lung and stomach carcinoma cells was accounted by NRP1-dependent EGFR upregulation [36].

### 3.3. Adhesion Molecules

#### 3.3.1. Integrins

**Figure 3:**
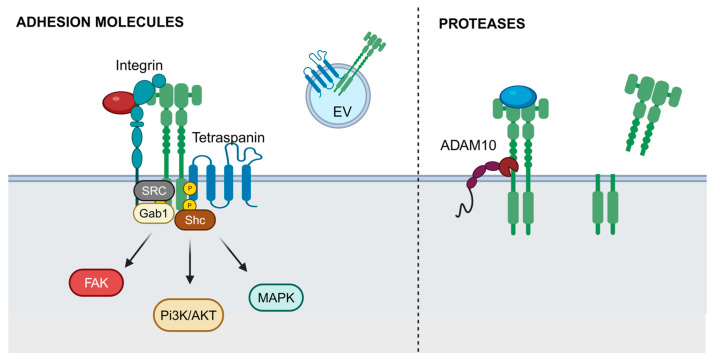
Cell surface proteins interacting with MET: Adhesion molecules (integrins, tetraspanins) and protease ADAM10. Created in BioRender (accessed on 18 December 2024).

Integrins are heterodimeric membrane receptors that mediate adhesive interactions between cells and their surrounding environment, including the extracellular matrix and adjacent cells [108,109]. They perform a scaffolding function by recruiting a complex network of proteins that link the actin cytoskeleton to extracellular components [110]. In addition to facilitating the tissue assembly with specific physical and mechanical properties, integrins play a critical role as molecular transducers, conveying extracellular signals to intracellular signaling pathways. Each integrin consists of an α and β subunit, resulting in a wide variety of integrin molecules, each interacting with specific ligands. This diversity supports their varied biological functions, including the regulation of the cell cycle, modulation of cell shape, motility, invasion, and metastasis. Their signaling pathways often intersect with those of MET. Based on various experimental models and results, it has been proposed that MET and integrin cooperation occurs through both inside-out and outside-in signaling (Figure 3) [37].

In the context of inside-out signaling, HGF-dependent MET activation leads to integrin activation and enhanced cell adhesion [44,45,53] and migration [38,39]. One mechanism involves increased integrin expression. For instance, HGF induces higher α9 integrin mRNA levels in lymphocytic endothelial cells [38]. Another mechanism involves increased phosphorylation of integrins, such as β4, in response to HGF stimulation [40]. A third potential mechanism involves HGF-mediated integrin localization, as seen with β1 integrin, which is directed to the basolateral membrane in human lung adenocarcinoma cells [45]. HGF also influences integrin intracellular trafficking, promoting β1 integrin’s internalization and subsequent recycling [46]. This trafficking is essential for cell invasion, with the clathrin adapter protein, huntingtin-interacting protein 1, playing a critical role in these processes. Knocking out the Rho GTPase ARF6 in mouse endothelial cells impairs HGF-dependent cell migration, spreading, and adhesion to β1 integrin ligands, such as collagen I and IV, and fibronectin [47]. This impairment also reduces HGF-stimulated β1 integrin recycling, crucial for angiogenesis. The ARF6 knockout or knockdown of its activator, the guanine exchange factor GRP1, significantly diminishes HGF-dependent angiogenesis and tumor growth in mice. These findings suggest that ARF6 influences angiogenesis and tumor progression through HGF-mediated β1 integrin recycling, although the downstream signaling mechanisms remain unclear.

In another scenario (outside-in signaling), the integrin binding to its extracellular ligand activates MET. Treating or plating ovarian, breast, lung, or prostate cancer cells on integrin substrates such as fibronectin, collagen, or laminin has been shown to trigger MET phosphorylation across various cell models [48,49,54,60,111]. MET phosphorylation decreases significantly when carcinoma cells are cultured in suspension but is restored upon the re-plating on substrates like fibronectin or collagen [111,112]. Moreover, increasing the concentration of fibronectin leads to higher levels of MET phosphorylation, highlighting the role of integrin interactions in modulating MET activity [48]. Silencing integrins α5, α5β1, β1, or β5 through siRNA, or using blocking antibodies against α5β1 or α5, significantly reduced or eliminated MET phosphorylation in vitro and in mouse xenografts [49,52,54,55,113,114]. Additionally, siRNA-targeting fibronectin also diminished MET phosphorylation. However, adding HGF, which induces MET phosphorylation, overcame the inhibition of an ovarian cancer cell invasion caused by α5β1 blocking antibodies or siRNA, indicating that integrins act upstream of MET in an HGF-independent manner, playing a crucial role in MET activation [54]. The exact mechanisms by which integrin signaling leads to MET phosphorylation are not fully understood. It is suggested that the cytoplasmic domain of integrins, particularly the β4 integrin, may influence MET phosphorylation. For instance, a truncated β4 integrin lacking key phosphorylation sites reduced MET phosphorylation [41]. Additionally, blocking integrin-linked kinase (ILK) or components of the Src-FAK (focal adhesion kinase) pathway can inhibit this phosphorylation, particularly in HGF-independent contexts [48,111]. The Src-FAK pathway has been identified as a central signaling route downstream of integrin-activated MET [54,60].

A third, less conventional mode involves integrins acting as signaling adaptors for MET, independent of their adhesive role, facilitating anchorage-independent survival [40,42,43,50,56]. Specifically, the MET/α6β4 integrin complex leads to the phosphorylation of the β4 integrin subunit, which subsequently activates Shc, PI3K, and Gab1 (Grb2 associated binding protein 1). Both Gab1 and α6β4 integrins serve as additional docking sites, facilitating the recruitment of more transducers that work alongside those directly linked to MET, thereby amplifying the signaling pathway [43]. The role of integrins, independent of their adhesive properties, was demonstrated by the fact that neither anti-β4 antibodies nor a β4 variant lacking its extracellular domain could block the invasion process. Additionally, the β4 integrin-MET interaction has been observed in endothelial cells, where HGF induces the formation of a complex involving the β4 integrin, MET, and the sphingosine 1-phosphate receptor 1 (S1PR1) at caveolin-enriched microdomains, enhancing vascular integrity [39]. Moreover, Barrow-McGee et al. (2016) [50] found that MET interacts with β1 integrin, that also functions as an adapter linking MET to Shc. Furthermore, this study revealed the co-internalization of both molecules. The internalized β1 integrin then associates with LC3B-positive autophagy-related endomembranes (ARE), promoting sustained ERK1/2 signaling. Metastatic cancer cells must survive without anchorage while traveling through the blood or lymphatic system. This inside-out cooperation between MET and β1 integrin, which functions independently of β1’s adhesive properties in non-adherent cells, may be utilized by these cells during transit. In such environments, the need for integrin-mediated adhesion is reduced, but there is an increased requirement for sustained signaling to protect against anoikis, a form of programmed cell death that occurs when cells detach from the extracellular matrix, preventing metastatic dissemination. A recent study found that two splice variants of the co-receptor neuropilin-1 enhance the interaction between MET and β1 integrin, leading to their co-internalization and accumulation in endosomes. This interaction provides sustained signals that activate the FAK/p130Cas pathway, promoting colorectal cancer cell migration, invasion, and metastasis [114]. Additionally, a computational model specific to hepatocellular carcinoma supports these findings, showing enhanced inhibition of phosphorylated AKT and ERK1/2 upon MET-α5β1 dissociation, highlighting the importance of MET-β1 co-trafficking [50,115]. Tumor cells exploit these receptor interactions to drive metastasis and resistance to invasive treatments [48]. A specific role for the MET/β1 integrin complex has been identified in breast cancer cell intravasation, showing a preferential affinity for bone type 1 collagen [51].

The proximity ligation assay indicates a likely direct interaction between MET and β1 integrin [48]. However, this association might involve additional molecules, particularly tetraspanin transmembrane proteins (see below), which facilitate cell-ECM interactions and integrin-RTK crosstalk. Indeed, integrin-mediated pathways are frequently linked to the development of drug resistance [116,117]. In mouse mammary tumor models, increased collagen levels, along with elevated β1 integrin and SRC activity, have been shown to contribute to combined resistance against HER2-targeted therapies (trastuzumab and pertuzumab) and anti-PI3K therapy (buparlisib) [116]. In lung cancer, β1 integrin–SRC–AKT is suggested to play a central role in acquired resistance to EGFR-targeted drugs [118]. Similarly, a β1 integrin–FAK–cortactin signaling network, involved in cellular adhesion and migration, has been identified as a mechanism that reduces the sensitivity of head and neck squamous cell carcinoma to radiotherapy [119]. Additionally, αvβ3 integrin is considered a potential marker for breast, lung, and pancreatic cancers with stem-like traits and high resistance to RTK inhibitors [120]. The involvement of β1 integrin and related signaling networks, such as SRC, AKT, and FAK, highlights the complexity of resistance mechanisms, particularly in response to targeted therapies and radiotherapy [121].

#### 3.3.2. Tetraspanins

MET has been found to interact with members of the tetraspanin superfamily (Figure 3), membrane-spanning proteins known for their strong association with integrins, potentially modulating their function through integrin compartmentalization [57]. This interaction suggests that certain tetraspanins can influence MET/integrin interactions and regulate MET signaling. Klosek et al. (2005) [58] demonstrated that in human salivary gland cells, CD151 associates with MET and α3 or α6 integrin subunits, enhancing HGF/MET signaling to promote cell migration and proliferation. Similarly, in MDA-MB-231 breast cancer cells, the CD151 interaction with MET triggers AKT activation, leading to branching networks in matrigel [59]. In GTL16 gastric carcinoma cells, Franco et al. (2010) [40] found that CD151 associates with the MET receptor, driving β4 integrin phosphorylation and facilitating the coupling of MET with Gab1–Grb2 (growth factor receptor-bound protein 2) to promote MAPK phosphorylation and tumor growth. Disrupting CD151 using siRNA interferes with these MET-integrin complexes, highlighting its role in their cooperation [40,58,59]. Conversely, several studies have shown that tetraspanin CD82 plays an inhibitory role in MET signaling, potentially in combination with gangliosides [60,61,62,63]. CD82 prevents MET phosphorylation upon HGF activation, exposure to extracellular matrix ligands, or EGFR transactivation, leading to the impaired binding of MET to Gab1/Grb2, reduced Ras or Src activation, and consequently decreased migration, invasion, or differentiation. Given the large family of tetraspanin proteins that interact with various integrins, it is likely that other tetraspanins also contribute to MET signaling. One potential candidate is CD9, which has been shown to promote cell migration, facilitate FAK phosphorylation, and interact with EGFR [122,123]. Tetraspanins play a significant role in the biogenesis, sorting, and function of extracellular vesicles (EVs). EVs are membrane-bound vesicles released by cells that transport biomolecules to other cells, facilitating intercellular communication. Tetraspanins are involved in the sorting of specific cargo into EVs and they play a crucial role in determining the destination and function of these vesicles. They can influence the incorporation of MET into EVs, affecting how MET is trafficked and potentially altering its signaling pathways. For instance, MET and its proteolytic fragments are sorted and secreted through a different pathway or type of EVs than those associated with CD81 [64]. Specifically, MET is more efficiently associated with high-density, detergent-insoluble EVs, while CD81 is enriched in low-density, detergent soluble EVs. This separation indicates that the mechanisms governing the sorting and secretion of MET are distinct from those involving CD81. The sorting of specific adhesion receptors into EVs via tetraspanins might influence the targeting of these EVs, as the inclusion of certain adhesion receptors and proteases in EVs relies on tetraspanin-enriched microdomains (TEMs). However, there are no studies directly investigating the role of TEMs in the precise targeting and uptake of MET-containing EVs by recipient cells.

### 3.4. Proteases

#### ADAM10

There is a known association between ADAM10 (A Disintegrin and Metalloproteinase 10) and the MET receptor (Figure 3), particularly regarding their roles in cellular processes such as signaling, migration, and cancer progression. ADAM10 is a membrane-bound metalloprotease involved in the cleavage and shedding of various cell surface proteins, modulating their activity. It cleaves the extracellular domains of these proteins, including receptors like MET and AXL. The interaction between ADAM10 and MET leads to the proteolytic processing of MET, which influences MET signaling [65,66,67]. Specifically, the ADAM10 cleavage of MET results in the release of its extracellular domain. This cleavage modulates MET’s activity and its ability to engage in signaling pathways. In the context of cancer, the shedding of MET by ADAM10 can affect tumor progression by altering how cells respond to growth factors and their surrounding environment. Moreover, the interactions between ADAM10 and MET could influence how these proteins are sorted into EVs [64], thereby impacting intercellular communication and the spread of signaling molecules in physiological and pathological processes, including cancer metastasis. The shedding of the MET receptor by ADAM10 serves as a negative feedback mechanism that modulates signaling activity, helping to maintain cellular homeostasis. However, in cancer, alterations in ADAM10 activity can lead to dysregulated signaling. For example, the reduced activity of ADAM10 can result in the accumulation of unprocessed RTKs on the cell surface, thereby enhancing proliferative and survival signals that contribute to cancer progression and resistance to therapies, such as kinase inhibitors [68].

### 3.5. Other Receptors

#### 3.5.1. CD44 Protein

**Figure 4:**
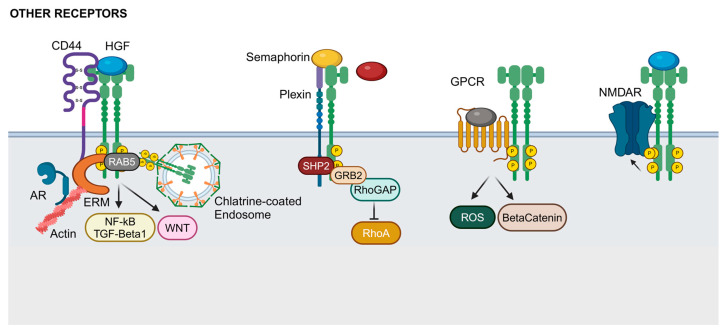
Other cell surface receptors interacting with MET: CD44, semaphorins and plexins, GPCRs, and NMDAR. Created in BioRender (accessed on 18 December 2024).

CD44 is a part of a family of transmembrane proteins derived from a single mRNA molecule that undergoes alternative splicing, producing multiple isoforms [124]. The interaction between MET and CD44 significantly promotes cancer progression and metastasis (Figure 4). 

CD44, a transmembrane glycoprotein receptor for hyaluronan, acts as a co-stimulator for MET, facilitating its activation and signaling [69]. Specifically, certain CD44 isoforms, like CD44v6, are essential for the HGF-dependent activation of MET [70]. This interaction supports various oncogenic processes, including cell proliferation, migration, and survival. Ezrin, Radixin, and Moesin (ERMs) are cytoskeletal linker proteins that connect cell surface receptors, such as MET and CD44, to the actin cytoskeleton, influencing cell shape and motility [125]. By interacting with ERM proteins, CD44v6 facilitates the internalization of MET into a rab5-positive endosomal compartment, where MET subsequently activates downstream signaling [126]. Several studies have confirmed that the overexpression of specific CD44 isoforms in various tumor cell systems enhances MET function and facilitates its interaction with NF-κB and TGF-β1 signaling pathways [71,127]. Although CD44v6 is a key co-receptor linking MET to ERM proteins, other molecules like ICAM1 can also fulfill this role in the absence of CD44v6 [128]. Other CD44 isoforms, such as CD44v9 in prostate cancer cells, stabilize the androgen receptor (AR) through MET signaling [129]. Additionally, CD44v10 has been implicated in MET-mediated vascular barrier enhancement by promoting its translocation to lipid rafts and recruiting essential scaffolding proteins [74]. Overall, various CD44 isoforms positively regulate MET by supporting its interaction with ERM proteins, linking it to other signaling pathways, and localizing it to caveolin-rich microdomains. Interestingly, MET activity in intestinal stem cells requires the co-expression of the CD44v4 isoform, a recognized target of the WNT pathway and a marker of both normal stem cells and cancer stem cells (CSCs) in various tissues [76,77]. In studies involving intestinal organoid cultures and mice with inducible MET deletion, HGF receptor signaling was found to play a crucial role in regulating intestinal homeostasis, regeneration, and adenoma formation [75]. These MET-related activities are supported by the CD44v4-10 isoform. The CD44v6 variant has also been shown to enhance invasion and metastasis in human colorectal cancer stem cells after transplantation into mice [130].

In several cancer types, including gliomas and colorectal cancer, this MET-CD44 interaction enhances the invasive potential of cancer cells and contributes to drug resistance [72,73]. For example, in gliomas, CD44 modulates MET signaling, primarily affecting cell proliferation without significantly altering survival pathways. In colorectal cancer, the specific collaboration of MET with CD44 suggests that targeting the MET-CD44v interaction could be a promising therapeutic strategy to mitigate the tumorigenic effects of MET signaling.

#### 3.5.2. Semaphorins and Plexins

Semaphorins are a large and diverse family of proteins that play crucial roles in axonal guidance, immune responses, and angiogenesis [131]. Semaphorins have a large extracellular SEMA domain that mediates the interactions with their receptors, Plexins [132]. This domain is structurally homologous to the SEMA domain found in MET and RON receptors. Plexins also possess an extracellular SEMA domain, facilitating the binding of semaphorins. Plexins are a diverse family of transmembrane receptors, segregated into four subfamilies, with significant roles in cancer cell behaviors such as proliferation, migration, and invasiveness. The interaction between MET and class B Plexins, especially Plexin B1, exemplifies the complexity of these signaling networks (Figure 4). MET can oligomerize with Plexin B1, which leads to the activation of the receptor by Semaphorin 4D (Sema4D), independently of its usual ligand, HGF [78,79]. This interaction enhances the cellular invasive activity when MET and Plexins are co-expressed [80]. Yet, this interplay is nuanced, as the balance of different receptor tyrosine kinases like MET and ErbB-2, which can both interact with Plexin-B1, can lead to opposing migratory responses [81]. In breast carcinoma cells, when Sema4D binds to Plexin-B1 associated with ErbB2, it induces cell migration and metastasis. Conversely, when Sema4D binds to Plexin-B1 associated with MET, it inhibits cell migration. This indicates that switching between these two receptor tyrosine kinases is enough to change Sema4D’s effect from promoting to inhibiting cell migration, and vice versa [81]. Similar observations were also reported in prostate cancer cells [82]. Cells only expressing Plexins respond to semaphorins with halted migration and collapse, indicative of a repulsive function unique to Plexins [133]. Accordingly, there are observations on an inhibitory role of Plexin B1 on MET function. In melanocytes and malignant melanoma cells, Sema4D inhibits HGF-induced MET activation, and reducing the Plexin B1 expression in these cells leads to MET activation rather than inactivation [83,84]. Plexin B1 and MET form a receptor–receptor complex in normal human melanocytes, with Sema4D enhancing this interaction [83]. Additionally, Sema4D boosts Shp2 (Src homology region 2-containing protein tyrosine phosphatase 2) expression, which is essential for HGF-dependent MAP kinase and AKT activation in melanocytes. This study suggests that Plexin B1 regulates MET signaling both directly, by inhibiting MET activation through receptor–receptor association, and indirectly, by modulating Shp2 levels, which control downstream MET-dependent signaling. In breast carcinoma cells, the Sema4D activation of Plexin B1 leads to its tyrosine phosphorylation by MET, creating a docking site for the SH2 domain of Grb2. Consequently, Grb2 is recruited into the Plexin B1 receptor complex and interacts with p190 RhoGAP via its SH3 domain, which mediates RhoA deactivation and subsequently inhibits the motility of breast carcinoma cells. Sema4D, highly expressed in various human tumors, can also induce angiogenesis through its interaction with Plexin B1 and MET in endothelial cells [85]. The source of Sema4D may vary across tumors; in some cases, tumor-associated macrophages, rather than the tumor cells themselves, are the primary producers [134]. Moreover, Sema3C has been identified as capable of activating a variety of tyrosine-kinase receptors, including MET [86]. In prostate cancer cells, monoamine oxidase A (MAOA) enhances the levels of Semaphorin 3C (Sema3C), NRP-1, and Plexin-A2. Sema3C engages with NRP-1 to form a signaling assembly that also involves Plexin-A2 and MET. This complex activates MET signaling pathways, which in turn promote the invasive growth of prostate cancer cells around nerve fibers [87].

#### 3.5.3. GPCRs

GPCRs (G-protein-coupled receptors) are a large receptor family mediating diverse cellular responses. GPCRs and RTKs were once thought to function as separate signaling entities, until groundbreaking research by Ullrich and colleagues [135] demonstrated that GPCR agonists activate tyrosine phosphorylation of EGFR. This process, known as transactivation, involves the modification of RTK activation and downstream signaling as a direct result of GPCR/RTK interactions. Transactivation provides a mechanism for expanding and diversifying the signaling networks within cells by integrating the wide range of GPCRs and their ligands with the extensive signaling pathways mediated by activated RTKs. The formation of oligomeric complexes between GPCRs and RTKs is now recognized as a key regulator of transactivation [88]. The MET receptor undergoes tyrosine phosphorylation not only in response to EGF stimulation but also upon activation by GPCR agonists (Figure 4) [89]. While the inhibition of EGFR kinase activity blocks EGF-induced MET activation, it does not prevent activation by GPCR agonists. This activation is linked to increased reactive oxygen species (ROS) levels in the cell. Moreover, the activation of the MET receptor by GPCR agonists, EGF, or its ligand HGF, leads to the release and nuclear translocation of beta-catenin, which is dependent on MET signaling. Yet, the mechanisms by which GPCRs and EGFR are connected to the oncogenic potential of MET signaling in cancer cells are not fully understood. Several mechanisms for GPCR-induced EGFR transactivation have been proposed. These include signaling pathways triggered by second messengers, such as intracellular Ca^2+^ release and protein kinase C (PKC) activation [136]. Non-receptor tyrosine kinases like Src can also phosphorylate EGFR directly or through adaptor proteins [137]. Another mechanism involves GPCR-induced cleavage of EGFR ligands via ADAMS metalloproteinases [138]. Additionally, direct interactions between GPCRs and EGFR have been observed, influencing EGFR phosphorylation and promoting invasive behaviors in cancer cells [139]. In prostate cancer cells, the pro-inflammatory chemokine receptor CXCR7 interacts with EGFR, and its overexpression leads to an increased phosphorylation of EGFR [140]. Metabotropic glutamate receptors (mGluR) transactivate EGFR family members in the central nervous system [141]. A recent study shows that mGluR1 directly interacts with and stabilizes the EGFR in a glutamate-dependent manner in lung cancer brain metastasis [142]. New therapeutic compounds will be developed that specifically target the interaction between mGluR1 and EGFR.

#### 3.5.4. NMDAR

NMDARs (N-methyl-D-aspartate receptors) are ion channels that play critical roles in synaptic plasticity in neurons and are implicated in cancer signaling. MET interacts with NMDAR in a way that significantly impacts neuronal function, particularly in synaptic plasticity and neuroprotection (Figure 4) [90]. These interactions between MET and NMDARs are crucial in both normal physiological conditions and in the context of neurological disorders. For instance, MET can regulate the trafficking and clustering of NMDARs at synapses, thereby affecting processes like long-term potentiation (LTP) and long-term depression (LTD), which are fundamental to synaptic plasticity. This regulation is important because the dysregulation of these processes is often associated with conditions such as Alzheimer’s disease and other neurodegenerative disorders. Furthermore, MET’s role in modulating NMDARs could also influence how neurons respond to excitotoxic stress, a condition where excessive glutamate causes the damaging overactivation of NMDARs, leading to neuronal injury or death [91]. While NMDARs are traditionally associated with neuronal tissues, they are also expressed in various cancer types (reviewed in Gallo [92]). HGF ligand stimulation induces MET clustering in complex with NMDAR on the cell surface of triple-negative breast cancer (TNBC) cells, a process that is impaired upon the pharmacological inhibition of NMDAR by MK801 and ifenprodil [93]. The signaling pathways activated by this interaction can enhance the invasive properties of cancer cells, contributing to tumor progression [93]. The synergistic activation of MET and NMDARs amplifies oncogenic signaling, leading to enhanced cell motility and the ability of cancer cells to invade surrounding tissues [94]. This synergy is particularly relevant in cancers where both receptors are overexpressed or hyperactivated. Targeting the MET-NMDAR interaction represents a potential therapeutic strategy in cancer treatment. By inhibiting this crosstalk, it may be possible to reduce tumor growth, metastasis, and resistance to existing therapies. This makes the MET-NMDAR complex a promising target for novel anti-cancer therapies.

## 4. Combination Therapies with Drugs Targeting MET and Its Interactors

The growing involvement of MET in cancer progression and therapeutic resistance has led to the development of numerous strategies targeting MET, establishing it as a promising candidate for novel anti-cancer treatments. While MET-targeting drugs show significant potential, they are not without limitations and have sometimes produced inconsistent clinical outcomes (reviewed in Gallo [143]). Continued research and clinical trials are essential to refine these strategies and ensure their effective translation into clinical practice. In this context, we focus on combinatory therapeutic approaches targeting MET and its cell surface interactors, aiming to overcome these limitations and enhance therapeutic efficacy.

### 4.1. Combination Therapies Involving MET and RTK Inhibitors

MET activation diminishes the effectiveness of TKIs due to the crosstalk between MET and RTK (particularly EGFR) signaling pathways [144,145]. When EGFR is activated, it can increase MET activation and vice versa. MET amplification bypasses EGFR blockage, allowing downstream signaling to continue and avoiding cell death, which makes it a key resistance mechanism against EGFR TKIs. This resistance occurs in about 5–21% of cases after first-line or second-line EGFR TKI therapy and increases to around 19% after resistance to osimertinib [20,146]. By simultaneously targeting both MET and EGFR, the combination therapy can block both pathways, preventing the crosstalk that drives resistance (Table 2) [147].

Combining MET inhibitors with EGFR inhibitors like gefitinib or erlotinib has shown enhanced efficacy in overcoming resistance in certain cancers [148,149,155,157,158,160,161,162]. Sequist et al. (2020) [150] evaluated the combination of osimertinib and savolitinib (a small-molecule MET tyrosine kinase inhibitor) in patients with EGFR-mutant NSCLC and MET amplification. The study found higher response rates and longer progression-free survival in patients who had not previously been treated with third-generation EGFR TKIs, particularly those with MET-driven resistance. These results suggest that osimertinib combined with savolitinib could be an effective option for patients with MET-driven resistance to EGFR TKIs.

The interaction between HER2 and MET plays a crucial role in explaining how MET overexpression drives resistance to HER2 inhibitors [164,165,166]. Trastuzumab, a monoclonal antibody targeting HER2, effectively blocks HER2 homodimerization but not heterodimerization with other RTKs. MET can form dimers with HER2, and this pairing is not inhibited by trastuzumab. Consequently, the HER2/MET interaction activates both receptors and their downstream signaling pathways, promoting cell invasion, particularly in epithelial cell models [22,167]. Strategies combining MET TKIs with HER2-targeted therapies (antibodies or TKIs) were initially tested in cell models. Research by Shattuck et al. (2008) [168] showed that inhibiting MET restored sensitivity to trastuzumab in HER2+ breast cancer cells. Similarly, in HER2-amplified gastric cancer cells that became resistant to afatinib, a TKI targeting the ErbB family of receptors, as a result of MET amplification, dual inhibition of HER2 and MET effectively suppressed tumor growth [166]. Combining lapatinib (a HER2-inhibitor) with foretinib (a MET and VEGFR inhibitor) also reversed resistance to lapatinib [153,154]. These findings highlight the potential of anti-MET strategies to overcome resistance to HER2-targeted therapies. However, a phase I study combining dacomitinib (a pan-HER inhibitor) with crizotinib (a small-molecule kinase inhibitor of MET and other RTKs) in advanced NSCLC patients showed limited success due to toxicity and modest tumor response [151].

MET and VEGFR signaling pathways are often co-activated in cancers and may work synergistically to promote tumor growth and resistance to therapy. In glioblastoma, a VEGF blockade disrupts the recruitment of a MET-inhibitory phosphatase (PTP1B) to the VEGFR2–MET complex, resulting in increased MET activation and tumor invasion [30]. Furthermore, anti-angiogenic therapies, which block VEGF signaling in tumor blood vessels, exacerbate tumor hypoxia, a condition of low oxygen levels that upregulates MET and activates MET-dependent invasive growth in cancer cells [169]. For these reasons, combination therapy involving MET inhibitors and VEGF inhibitors have been used in preclinical models and in patients. Targeting MET alongside VEGF inhibition with the small-molecule kinase inhibitor, cabozantinib, can reduce tumor aggressiveness in pre-clinical pancreatic and neuroblastoma cancers [170,171]. Cabozantinib is also used in patients’ thyroid and renal cancers [172,173]. Multi-target MET TKIs that target both MET and an additional RTK, such as merestinib or glesatinib, can greatly enhance efficacy and address some of the limitations associated with single-target inhibitors, such as drug resistance [174]. Bevacizumab, a monoclonal antibody that targets VEGF-A, inhibits angiogenesis. When combined with MET inhibitors, the aim is to simultaneously block both angiogenesis and MET-driven tumor survival and pro-invasive mechanisms. In NSCLC, MET-driven resistance to bevacizumab has been observed, and MET inhibitors could potentially restore sensitivity to anti-VEGF therapy [159,175]. Moreover, the combination of axitinib, a VEGFR inhibitor, plus crizotinib has shown evidence of antitumor activity in metastatic renal cell carcinoma [152]. Ramucirumab is a monoclonal antibody that targets the VEGFR2, blocking its activation and thereby inhibiting angiogenesis [163]. The rationale for combining MET inhibitors with ramucirumab is similar to that with bevacizumab, though there are some differences due to the specific targeting of VEGFR2 rather than VEGF-A [156,163].

### 4.2. Disrupting MET-Driven Metastasis

MET overexpression in tumors can arise not only from an increased transcription within individual cells but also from the expansion of MET-expressing cells over non-expressing ones. In tumors, there is often a shift toward an abundance of stem and progenitor cells, with fewer differentiated cells, a reversal of the composition in normal tissues, where differentiated cells predominate. Since MET is typically expressed in stem and progenitor cells, its widespread presence in tumors is a marker of CSCs. These CSCs, often transformed from normal stem cells, use MET signaling to maintain stem-like functions essential for tumor initiation, growth, regeneration, and spread. MET and EGFR are known to cooperate in various tissues to sustain stem cell self-renewal and support CSC populations. This interaction can enhance these processes, particularly in cancer, where it promotes tumor growth and resistance to therapies. This cooperation is observed in multiple tissue types, including lung, brain, and other epithelial-derived cancers, and has significant therapeutic implications. For instance, colorectal cancers with an intact RAS pathway often lack prominent oncogenic drivers and rely heavily on CSCs that depend on growth factors such as EGF, FGF, and HGF for their proliferation and survival [176]. This highlights the critical role these growth factors play in regulating CSC properties and sustaining tumor growth and progression.

MET is frequently observed to be highly expressed in metastatic uveal melanoma, where it is associated with poor prognosis, and plays a role in treatment resistance mechanisms [177,178]. Notably, a combination therapy with crizotinib and darovasertib, a PKC inhibitor, has demonstrated a manageable safety profile and clinical efficacy superior to current standards of care for metastatic uveal melanoma carrying GPCRs driver mutations [177,178,179].

The functional interaction between MET and CD44 plays a key role in the dissemination of CSCs, a critical step in metastases development. This cooperation facilitates the migration and invasion of CSCs, enabling their spread and the formation of secondary tumors [180]. Studies have shown that targeting CD44v6 can effectively inhibit MET signaling [130,181]. The HGF/MET/CD44v6 signaling pathway promotes tumor growth and metastasis in pancreatic cancer. In several mouse models, blocking CD44v6 with a specific peptide effectively halted tumor progression and metastasis [181]. Notably, when compared to crizotinib, the CD44v6 peptide showed a stronger effect, suggesting that inhibitors with a broader activity may have greater therapeutic potential. Two peptides identified from a phage-display peptide library were found to inhibit MET activation by targeting CD44v6 [182]. These peptides also suppressed metastasis in both human and mouse breast cancer cells, demonstrating effectiveness in experimental and spontaneous metastasis models.

The formation or disruption of the MET-integrin complex has significant functional implications for cancer cell behavior. This was demonstrated using an engineered heterodimerization system in which the rapamycin-derived drug AP21967 induced the dimerization of MET-FKBP and β1 integrin-FRB in breast cancer cells. This artificial dimerization promoted enhanced wound healing and invasion, highlighting the functional relevance of the MET-β1 complex in tumor progression. Additionally, a therapeutic humanized anti-β1 neutralizing antibody inhibited MET-β1 interaction in breast cancer cells, further reducing cell motility and invasion [48]. These findings suggest that targeting MET-integrin interactions may have therapeutic potential for preventing cancer invasion and metastasis. However, this interaction may not occur in isolation. Tetraspanin transmembrane proteins, which facilitate cell-ECM interactions and integrin-growth factor receptor crosstalk, could mediate the cooperation between integrins and MET. Specifically, CD151 has been identified as a part of MET and integrin complexes, such as β4 or α3/α6 integrins, in gastric carcinoma and salivary glands, and breast cancer cells [40]. The loss of CD151 through siRNA knockdown disrupted these MET–integrin complexes, underscoring its role in maintaining their association [58]. These findings suggest a broader, more complex network of molecules that facilitate integrin–MET interactions and contribute to cancer progression.

## 5. Conclusions

The MET receptor interacts with a wide array of surface plasma membrane proteins, including other receptor tyrosine kinases (e.g., EGFR, HER2, HER3, and VEGFR2), co-receptors (e.g., CDCP1 and Neuropilin-1), cell adhesion molecules (e.g., plexins, integrins, and CD44), and synaptic receptor NMDAR. These interactions are pivotal in regulating diverse cellular processes such as proliferation, survival, migration, invasion, and angiogenesis. In the context of cancer, the MET interactome contributes to tumor progression, metastasis, and therapeutic resistance, making these interactions attractive targets for the development of novel anti-cancer therapies.

## Figures and Tables

**Table 1:** MET-interacting proteins and complex functions.

Interacting Protein			Cell System	Complex Functions	References
Receptor Tyrosine Kinases		RON	NIH3T3, MET-amplified cancer cells, CRC cells, TNBC cells	Transphosphorylation, Proliferation, Tumorigenesis, Chemoresistance	[7,8,9,10]
		ROR1	COS-7, MET-amplified LC cells	Phosphorylation, Proliferation, Protection from apoptosis, Cell invasion	[11,12]
		EGFR	MET-amplified LC cells, Metastatic HNC, CRC cancer, NSCLC	Transphosphorylation, Protection from apoptosis, Drug resistance	[13,14,15,16,17,18,19,20,21,22,23,24,25]
		HER2	MET-amplified LC cells, NSCLC	Transphosphorylation, Protection from apoptosis, Cell migration, Drug resistance	[22,25]
		HER3	MET-amplified LC cells, HNC	Transphosphorylation, Protection from apoptosis, Drug resistance	[13,19,25,26,27,28]
		RET	MET-amplified lung cancer cells	Transphosphorylation, Cell migration	[25]
		IGF1R	PC cells	Cell migration and invasion	[29]
		VEGFR	GBM cells	Inhibition of MET by PTP1B and tumor cell migration	[30]
		NTRK3	In silico analysis	NA	[31]
		PDGFRβ	In silico analysis	NA	[31]
		INSR	In silico analysis	NA	[31]
		TYRO3	In silico analysis	NA	[31]
Co-Receptors		CDCP1	Carcinoma cell lines, LC cells, TNBC cells	EGFR-targeted drug resistance, Cell migration and invasion, EMT	[32,33,34]
		Neuropilin-1	PC cells, MET-addicted stomach and LC cells	Cell invasion, Drug resistance	[35,36]
Adhesion Molecules	Integrins	α9 Integrin	Lymphocytic endothelial cells	MET-mediated stimulation of integrin mRNA expression	[37,38]
		β4 Integrin	Prostate cancer cells, Endothelial cells	MET-mediated integrin phosphorylation, Integrin-dependent MET phosphorylation, Vascular integrity	[37,39,40,41,42,43]
		β1 Integrin	LC cells, Endothelial cells, CRC cells, BC cells, NSCLC cells	MET-mediated integrin relocalization and internalization, Integrin-dependent MET phosphorylation, Cell migration, spreading, invasion and metastasis, Angiogenesis, Drug resistance	[37,44,45,46,47,48,49,50,51]
		α5 Integrin	Metastatic ovarian cancer cells	Integrin-dependent MET phosphorylation, Drug resistance	[37,52]
		α5β1 Integrin	Ovarian cancer, Lymphoma cells	Integrin-dependent MET phosphorylation, Cell invasion and metastasis	[37,53,54]
		β5 Integrin	Oral squamous cell carcinoma	Integrin-dependent MET phosphorylation, Cancer stemness, Drug resistance	[37,55]
		α6β4 Integrin		Docking site for MET signaling, Invasive growth	[37,56]
	Tetraspanins	CD151	Salivary gland cells, TNBC cells, GC cells	HGF/MET signaling stimulation, MET/Integrin interaction, Cell migration, Proliferation, and invasion	[40,57,58,59]
		CD82	Oligodendrocytes, Ganglioside	HGF/MET signaling inhibition	[57,60,61,62,63]
		CD81		MET EV sorting	[57,64]
Proteases		ADAM10	MET-addicted cancer cells	MET shedding, Inhibition of cancer progression, Drug resistance, EV sorting	[64,65,66,67,68]
Other Receptors	CD44	CD44v6	Melanocytes, LC cells, CRC cells, GBM	Receptor co-stimulation, HGF-dependent MET activation, MET endosome internalization, ERM proteins interaction, Cancer progression and metastasis, Drug resistance	[69,70]
		CD44v9	Prostate cancer cells, GBM, CRC	Receptor co-stimulation, Cancer progression and metastasis, Drug resistance	[69,71,72,73]
		CD44v10	Intestinal adenoma, GBM, CRC	Receptor co-stimulation, Vascular barrier enhancement, Cancer progression and metastasis, Drug resistance	[69,72,73,74,75]
		CD44v4	Cancer stem cells, Intestinal adenoma, GBM, CRC	Receptor co-stimulation, Cancer progression and metastasis, Drug resistance	[69,72,73,75,76,77]
	Plexins	Plexin B1	BC cells, Prostate cancer cells, Ovary tumors, Melanocytes, Malignant melanoma cells, BC cells	HGF-independent receptor stimulation, Cell invasion, Inhibition of MET receptor and cell motility, Angiogenesis	[78,79,80,81,82,83,84,85]
		Plexin A2	Prostate cancer cells	HGF-independent receptor stimulation, Invasive growth	[86,87]
	GPCRs	GPCRs	Carcinoma cells	Receptors transactivation	[88,89]
	NMDAR	NMDAR	Neurons, TNBC cells	Neuroprotection, Synaptic plasticity, Cancer invasion	[90,91,92,93,94]

BC: breast cancer; CRC: colorectal cancer; EMT: epithelial mesenchymal transition; EV: extracellular vesicle; GBM: glioblastoma; HNC: head and neck cancer; LC: lung cancer; LTD: long-term depression; LTP: long-term potentiation; NSCLC: non-small cell lung cancer; NA: not available; PC: pancreatic cancer; TNBC: triple-negative breast cancer.

**Table 2:** MET-targeting agents and treatment combinations.

MET Inhibitor	Combined with	Target	Tumor	References
Tepotinib	Gefitinib	MET-EGFR	NSCLC	[148]
Capmatinib	Erlotinib	MET-EGFR	NSCLC	[149]
	Cetuximab	MET-EGFR	CRC	[147]
Savolitinib	Osimertinib	MET-EGFR	NSCLC	[150]
Crizotinib	Dacomitinib	MET-HER	NSCLC	[151]
	Axitinib	MET-VEGFR	Metastatic RCC	[152]
Foretinib	Lapatinib	MET-HER2	Metastatic breast cancer, Esophageal adenocarcinoma	[153,154]
	Erlotinib	MET-EGFR	NSCLC	[155]
Merestinib	Ramucirumab	MET-VEGFR	Metastatic CRC	[156]
Glesatinib	Erlotinib	MET-EGFR	Advanced solid tumors	[157]
Onartuzumab	Erlotinib	MET-EGFR	NSCLC	[158]
	Bevacizumab	MET-VEGF	NSCLC	[159]
Emibetuzumab	Erlotinib	MET-EGFR	NSCLC	[160,161,162]
	Ramucirumab	MET-VEGFR	Advanced solid tumors	[163]

CRC: colorectal cancer; NSCLC: non-small-cell lung cancer; RCC: renal-cell carcinoma.

## Abbreviations

ADAM10, a Disintegrin and Metalloproteinase 10; ARE, autophagy-related endomembranes; C-tail, C-terminal tail; CAV1, caveolin-1; CDCP1, CUB domain-containing protein; CSCs, cancer stem cells; Cryo-EM, Cryo-electron microscopy; ECD, extracellular domain; EGFR, epidermal growth factor receptor; EMT, epithelial-mesenchymal transition; ERM, Ezrin, Radixin, Moesin; EVs, extracellular vesicles; FAK, focal adhesion kinase; GPCRs, G protein-coupled receptors; Gab1, Grb2 associated binding protein 1; Grb2, growth factor receptor-bound protein 2; HNC, head and neck cancer; HER, human epidermal growth factor receptors; HGF, hepatocyte growth factor; GBM, glioblastoma multiforme; IGF1R, insulin-like growth factor-I receptor; ILK, integrin-linked kinase; INSR, insulin receptor; IPT, immunoglobulin–plexin–transcription factor domain; JM, juxtamembrane domain; K, kringle domain; KD, kinase domain; LTD, long-term depression; LTP, long-term potentiation; MAOA, monoamine oxidase A; mGluR, metabotropic glutamate receptors; N, N-terminal; NMDARs, NMDA receptors; NRP1, Neuropilin-1; NSCLC, non-small cell lung carcinoma; NTRK3, neurotrophic receptor tyrosine kinase 3; PDGFRβ, platelet-derived growth factor receptor β; PKC, protein kinase C; PRD, proline-rich domain; PSI, plexin–semaphorin–integrin domain; PTP1B, protein tyrosine phosphatase 1B; RET, rearranged during transfection receptor; RON, Recepteur d’Origine Nantais; ROR1, Receptor Tyrosine Kinase-Like Orphan Receptor 1; ROS, reactive oxygen species; RTKs, receptor tyrosine kinases; S1PR1, sphingosine 1-phosphate receptor 1; SEMA, semaphorin-like domain; Sema3C, Semaphorin 3C, Sema4D, semaphorin 4D; SFK, SRC family kinase; Shp2, (Src homology region 2-containing protein tyrosine phosphatase 2); SPH, serine protease homology domain; TEMs, tetraspanin-enriched microdomains; TKIs, tyrosine kinase inhibitors; TYRO3, tyrosine-protein kinase receptor; TM, transmembrane domain; TNBC, triple-negative breast cancer; VEGFR, VEGF receptor.
